# Ginsenoside Rb3 Suppresses Peste des Petits Ruminants Virus Replication by Inhibiting Autophagy to Potentiate Immune Responses

**DOI:** 10.3390/microorganisms14040738

**Published:** 2026-03-26

**Authors:** Qinglu Zhao, Hongmei Chen, Zhanying Hu, Dingcheng Wei, Xueliang Zhu, Rui Zhang

**Affiliations:** 1 Key Laboratory of Animal Medicine of Sichuan Education Department, Southwest Minzu University, Chengdu 610041, China; zql3278153307@163.com (Q.Z.); 15775474133@163.com (H.C.); 18848260360@163.com (Z.H.); wdc18111950049@163.com (D.W.); 2 State Key Laboratory for Animal Disease Control and Prevention, Lanzhou Veterinary Research Institute, Chinese Academy of Agricultural Sciences, Xujiaping 1, Yanchangpu, Lanzhou 730046, China; zhuxueliang@caas.cn

**Keywords:** Peste des petits ruminants virus, ginsenoside Rb3, antiviral, autophagy, immunomodulatory strategy

## Abstract

Peste des Petits Ruminants (PPR), a highly contagious disease of domestic and wild small ruminants, is characterized by severe morbidity and mortality. PPRV, the causative agent, is a *morbillivirus* in the family *Paramyxoviridae*. The virus poses a significant barrier to sustainable agricultural development in the developing world. Currently, no effective therapeutics agent for PPRV infection is available. Ginsenoside Rb3, the major bioactive constituent in the plants of ginseng, was reported to exert a wide range of pharmacologic and immunologic effects. However, it is unclear whether Ginsenoside Rb3 can act as an antiviral against PPRV infection. Here, we show that Ginsenoside Rb3 exhibits significant antiviral activity against PPRV in cell culture models. The mechanism of action of Ginsenoside Rb3 against PPRV is mainly attributed to its ability to inhibit PPRV-mediated autophagy, thus leading to promotion of interferon responses. In summary, our study establishes Ginsenoside Rb3 as a novel antiviral agent effective against PPRV, sheds light on its mode of action, and reveals a novel immunomodulatory strategy that may prove essential for combating both current and future viral outbreaks.

## 1. Introduction

Peste des petits ruminants (PPR), also known as ‘goat plague’, ‘kata’, ‘ovine rinderpest’, or ‘stomatitis-pneumoenteritis syndrome’, is an acute and highly contagious viral disease that primarily affects domestic sheep and goats, while also posing a threat to wild small ruminants. The disease is endemic across extensive regions of Africa, the Middle East, and Asia and is increasingly recognized as an emerging threat in new geographical areas [1]. PPR is characterized by high morbidity and mortality rates, which can reach up to 90% in naïve populations [2], leading to severe socioeconomic losses and threatening food security and the livelihoods of farmers in affected regions [3]. PPR is currently regarded as one of the fastest-spreading and potentially the most economically important diseases of small ruminants in the developing world—where these animals are integral to agricultural systems and rural development [4]. Due to its substantial impact on animal health and sustainable agriculture, PPR has been targeted by the World Organisation for Animal Health (WOAH) and the Food and Agriculture Organization (FAO) for global eradication by 2030 [5].

Peste des petits ruminants virus (PPRV), the etiological agent of PPR, is an enveloped, negative-sense, single-stranded RNA virus classified within the genus *Morbillivirus* of the family *Paramyxoviridae* (subfamily *Paramyxovirinae*, order *Mononegavirales*) [6,7]. This genus encompasses several highly impactful veterinary viral pathogens, such as rinderpest virus (RPV), canine distemper virus (CDV), marine morbilliviruses phocine distemper virus (PDV), dolphin morbillivirus (DMV) and porpoise morbillivirus (PMV), together with the only human measles virus (MV) [8,9]. Notably, the genus continues to expand, with recent characterizations of novel members such as Feline morbillivirus [10] and numerous morbilli-like viruses identified in bats and rodents [11]. A hallmark of PPRV pathogenesis, consistent with that observed for other morbilliviruses, is the induction of a severe yet transient state of immunosuppression [12,13]. This condition significantly heightens susceptibility to opportunistic secondary infections, thereby critically influencing disease severity and mortality [12,14]. The underlying immunosuppression results from a combination of direct viral cytopathic effects within lymphoid and epithelial cells and the deployment of multiple immune-evasive strategies by the virus [13,15], which collectively impair both innate and adaptive host immune responses. Thus, there is an urgent need to develop safe and effective antiviral agents against PPRV.

At present, the prevention and control of PPR primarily relies on vaccination, with live attenuated vaccines PPRV Nigeria75/1 (lineage II) and PPRV Sungri 96 (lineage IV) being the most widely used and extensively validated options [16,17,18,19]. However, these vaccines face substantial practical limitations, particularly in endemic regions. As a paramyxovirus, PPRV is inherently heat-sensitive [16,20], necessitating a continuous cold chain for vaccine distribution—a requirement that is difficult to meet in many PPR-affected countries, which are typically located in hot climates with underdeveloped infrastructure and unreliable electricity supplies. In addition, the high production cost of live attenuated vaccines further constrains their accessibility and large-scale deployment. Although sanitary measures such as movement restrictions and stamping-out policies can theoretically help contain outbreaks, their implementation remains economically and logistically challenging in most endemic settings, which are predominantly in developing countries [16]. Compounding these challenges is the fact that no antiviral drugs have yet been approved for the treatment of PPRV infection. Therefore, there is a pressing need to develop safe, effective, and thermostable antiviral agents to complement vaccination and enhance PPR control strategies.

Ginseng, a traditional herbal medicine that has been widely used in Asian medicine [21,22,23]. It contains numerous pharmacologically active substances, including polysaccharides, polyacetylenes, phytosterols, and essential oils, with ginsenosides being recognized as the major bioactive compounds [24]. These components confer a broad spectrum of biological and immunomodulatory activities, such as anti-inflammatory [25], antioxidant [26], modulate immune system [27], preventing mutagenesis [28] and cancer [29], and showing potential in alleviating conditions like colitis [30] and diabetes [31]. Although fermented ginseng extracts and several Ginsenosides have demonstrated broad biological effects—from antioxidative to antidiabetic—there are few reports about the protective effect of Ginsenosides on viral infection. One of the compounds, Ginsenoside Rg3, exhibited the notable and promising anti-Hepatitis C virus (HCV) potential [32]. Ginsenosides Rb1 and Rg1 have been shown to restrict murine norovirus (MNV) replication in vitro [33]. Ginsenoside Rb2 and Rb3 have been demonstrated to inhibit bovine viral diarrhea virus (BVDV) and classical swine fever virus (CSFV) in vitro [34]. Furthermore, fermented ginseng extracts showed promising antiviral activity against a broad range of influenza viruses [35]. Despite the documented antiviral role of Ginsenosides [35,36,37], its ability to inhibit PPRV infection has not yet been investigated.

The innate immune system detects viral nucleic acids through specific cytosolic sensors [38]: retinoic acid-inducible gene I (RIG-I) and melanoma differentiation-associated gene 5 (MDA5) function as principal sensors for viral RNA [15]. Upon detection of viral RNA, RIG-I and MDA5 activate by engaging the mitochondrial antiviral signaling protein (MAVS). Subsequently, MAVS recruits and activates TANK-binding kinase 1 (TBK1), which phosphorylates both MAVS itself and the transcription factor interferon regulatory factor 3 (IRF3). Phosphorylated IRF3 dimerizes and translocates to the nucleus, where it drives the expression of type I interferons (IFN-I) [38,39]. Secreted IFN-I signals in an autocrine or paracrine manner by binding to cell surface receptors, thereby activating the JAK/STAT pathway and leading to the induction of hundreds of interferon-stimulated genes (ISGs), collectively establishing an antiviral cellular state [40,41]. In parallel, MAVS-mediated signaling can also activate the IkB kinase (IKK) complex, leading to the phosphorylation and degradation of IkB proteins, thus releasing transcription factors of the nuclear factor κB (NF-kB). Subsequently, NF-κB translocates to the nucleus and induces the production of pro-inflammatory cytokines such as interleukin-6 (IL-6) and tumor necrosis factor-α (TNF-α), thereby enhancing inflammatory responses during viral infection [42,43].

Autophagy, an evolutionarily conserved intracellular lysosomal degradation pathway, serves as a critical mechanism for maintaining cellular homeostasis [44,45]. It facilitates the clearance of damaged organelles, misfolded proteins, and invading microorganisms, thereby protecting cells from metabolic stress, nutrient deprivation, and infection [46,47,48]. Morphologically, autophagy is characterized by the formation of a double-membrane vesicle, the autophagosome, which engulfs cytoplasmic cargo and delivers it to lysosomes for degradation and recycling. This highly regulated process involves three main stages (initiation, elongation, and maturation) orchestrated by over 20 conserved autophagy-related (ATG) proteins that assemble at specific endoplasmic reticulum subdomains upon induction. Key molecular markers for monitoring autophagy include microtubule-associated protein 1 light chain 3 (LC3), which is widely used to track autophagosome formation [49]. Beyond its role in cellular quality control, autophagy also plays an integral part in innate and adaptive immunity, participating in host defense against diverse intracellular pathogens such as bacteria, viruses, and protozoa [50,51]. An increasing amount of evidence has shown that the autophagy can be exploited by PPRV to facilitate its replication [15,52,53,54]. However, the effects of Ginsenoside Rb3 on morbilliviruses replication and the association between Ginsenoside Rb3 and autophagy have not yet been investigated.

The objective of this study was to assess the impact of Ginsenoside Rb3 on PPRV replication and to investigate its mechanism of action in vitro. The resulting data uncover key molecular pathways modulated by Ginsenoside Rb3, which could contribute to the design of novel interventions for controlling PPRV and potentially related viruses such as MV.

## 2. Materials and Methods

### 2.1. Cell Culture and Virus Propagation

African green monkey kidney (Vero) cells (ATCC, CCL-81TM) were cultured in Dulbecco’s modified Eagle’s medium (DMEM, Gibco, Carlsbad, CA, USA) supplemented with 10% heat-inactivated fetal bovine serum (FBS, Gibco) and penicillin-streptomycin solution (100 U/mL and 100 µg/mL, respectively). Caprine endometrial epithelial (EEC) cells were kindly provided by Prof. Yongxi Dou (Lanzhou Veterinary Research Institute, Chinese Academy of Agricultural Sciences, Lanzhou, China) and cultured in Dulbecco’s minimal essential medium/F-12 Ham’s medium (DMEM/F12) supplemented with 10% foetal bovine serum (FBS, Gibco), 100 IU/mL penicillin, and 10 μg/mL streptomycin. These cells were cultured as monolayers in cell culture flasks or dishes at 37 °C in a humidified atmosphere of 5% CO_2_ in air.

The PPRV attenuated vaccine strain Nigeria 75/1 was obtained from our laboratory’s culture collection. The viral stock was generated by infecting monolayers of Vero cells. PPRV was inoculated into Vero cells and cultured in DMEM supplemented with 2% FBS at 37 °C with 5% CO_2_ for 5 days until a obvious cytopathic effect (CPE) was observed in approximately 80% of the cells. The virus-containing media were collected, and the cells were lysed by three freeze–thaw cycles.

Virus titers were determined by inoculating cell monolayers in 96-well plates with 10-fold serial dilutions of viral stock, followed by incubation at 37 °C for 5–7 days. The 50% tissue culture infective dose (TCID_50_)/mL was calculated using the Reed and Muench method. The multiplicity of infection (MOI) for each experiment was based on the calculated titer for the respective cell line.

### 2.2. Antibodies and Reagents

For Western blotting, the following antibodies were used: Rabbit anti-GAPDH (5174), rabbit anti-LC3B (2775), rabbit anti-SQSTM1/p62 (5114), rabbit anti-ATG16L1 (8089), rabbit anti-Phospho-IRF3 (29047), rabbit anti-IRF3 (11904), rabbit anti-Phospho-TBK1 (5483), rabbit anti-TBK1 (3504), rabbit anti-Phospho-NF-kB p65 (3033), and rabbit anti-NF-kB p65 (8242) were from Cell Signaling Technology (Danvers, MA, USA). Mouse monoclonal antibodies against the N protein of PPRV were obtained from the Lanzhou Veterinary Research Institute, Chinese Academy of Agricultural Sciences, Lanzhou, China. Anti-rabbit IgG, HRP-linked antibody (CST, 7074, Danvers, MA, USA) and Anti-mouse IgG, HRP-linked antibody (CST, 7076, Danvers, MA, USA). Ginsenoside Rb3 (HY-N0041) and Chloroquine (HY-17589A) were purchased from MCE (South Brunswick Township, NJ, USA). Lipofectamine 2000 (11668019) was purchased from Thermo Fisher Scientific (Grand Island, NE, USA).

### 2.3. Cytotoxicity Assay

Cytotoxicity was evaluated using a Cell Counting Kit-8 (CCK-8) assay (Absin, abs50003, Shanghai, China). Briefly, the cells were seeded in a 96-well plate and cultured for 24 h. Subsequently, the culture medium was replaced with 100 µL of fresh medium containing Ginsenoside Rb3(MCE, HY-N0041) at various final concentrations (0, 10, 20, 30, and 40 µM). After a 48 h incubation at 37 °C under 5% CO_2_, 10 µL of CCK-8 solution was added to each well, followed by a further 2 h incubation at 37 °C. The optical density (OD) at 450 nm was then measured for each set of quadruplicate wells using an ELISA microplate reader (PerkinElmer, VICTOR NivoTM, Waltham, MA, USA).

### 2.4. Drug Treatment

The cells were seeded into new culture flasks or dishes. At the time of seeding, Ginsenoside Rb3 was added to the culture medium at 40 µM. The cells were either mock-infected or infected with Peste des Petits Ruminants Virus (PPRV) at 24 h after seeding with MOI of 1. The virus inoculum was removed after a 1 h adsorption period at 37 °C under 5% CO_2_, and the cells were washed to remove unbound viral particles. Subsequently, the cells were cultured in fresh DMEM containing 2% FBS, with the respective concentration of Ginsenoside Rb3 maintained in the treatment groups. Both cells and corresponding culture supernatants were harvested at 48 h post-infection (hpi) for subsequent analysis.

### 2.5. Western Blotting

The cells, either treated with 40 µM Ginsenoside Rb3 or left untreated, were harvested at 48 hpi. After washing with cold PBS, cells were lysed in RIPA buffer supplemented with protease and phosphatase inhibitors. Lysates were centrifuged to clarify, and protein concentrations were quantified using a BCA assay. Equal amounts of protein were denatured, separated by SDS-PAGE (10% gel), and transferred to PVDF membranes. Following blocking with 5% non-fat milk, membranes were probed overnight at 4 °C with primary antibodies. After washing, incubation with HRP-conjugated secondary antibodies was performed at room temperature for 1 h. Immunoreactive bands were visualized using an ECL substrate. GAPDH was used as a loading control. Images were acquired using a digital imaging system.

### 2.6. Quantitative Real-Time PCR

At 48 hpi, total RNA was isolated from cells treated with or without 40 µM Ginsenoside Rb3 using the RNeasy Plus Universal Mini Kit (Qiagen, Hilden, Germany, 73404). First-strand cDNA was synthesized from the RNA using Maxima H Minus cDNA synthesis master mix (Thermo Scientific, M1682). Quantitative real-time PCR (qPCR) was then performed using PowerUp SYBR Green Master Mix (Applied Biosystems, Waltham, MA, USA, 1801040), with cycling conditions set as per the manufacturer’s protocol. The live-attenuated PPRV Nigeria75/1 vaccine strain served as a positive control. The primers used were as follows:

PPRV N forward: 5′-AGAGTTCAATATGTTRTTAGCCTCCAT-3′
PPRV N reverse: 5′-TTCCCCARTCACTCTYCTTTGT-3′
GAPDH forward (Vero): 5′-CGAGATCCCTCCAAAATCAA-3′
GAPDH reverse (Vero): 5′-TGACGATCTTGAGGCTGTTG-3′
ATG16 forward (EEC): 5′-GGACATGATGGTTCGTGGAA-3′
ATG16 reverse (EEC): 5′-GGTCAATCACCAACTGGGCTA-3′
IFN-β forward (EEC): 5′-TGCCTCCTCCAGATGGTTCT-3′
IFN-β reverse (EEC): 5′-TGACCAATACGGCATCTTCC-3′
ISG15 forward (EEC): 5′-GAAGCAGTTCATCGCCCAGA-3′
ISG15 reverse (EEC): 5′-ACCTCATAGGAGCTGCTGCG-3′
IL-6 forward (EEC): 5′-TTCCAATCTGGGTTCAATCA-3′
IL-6 reverse (EEC): 5′-TTTCCCTCAAACTCGTTCTG-3′
IL-1β forward (EEC): 5′-ATGCTTCCAATCTGGGTTCA-3′
IL-1β reverse (EEC): 5′-ATGCTTCCAATCTGGGTTCA-3′
GAPDH forward (EEC): 5′-CCACGAGAAGTATAACAACACCC-3′
GAPDH reverse (EEC): 5′-GGTCATAAGTCCCTCCACGAT-3′

### 2.7. RNA Interference

The siRNAs target ATG16 gene of EEC cells were synthesized by Sangon Biotech (Shanghai, China). Scrambled siRNA was used as a negative control. The silencing efficiency was measured by quantitative Real-Time PCR. SiRNAs sequences are shown in the following:

siRNA-ATG16 sense: 5′-GCUGAGAAUUAAACACCAAGA-3′
siRNA-ATG16 antisense: 5′-UUGGUGUUUAAUUCUCAGCUG-3′

For construction of lentiviral CRISPR-Cas9 vectors, gRNAs are designed using gRNA Designer from Feng Zhang’s lab. Primers are synthesized and cloned into Lenti CRISPR v2 vector by ligation. The target sequence of ATG16 gene is: 5′-ACAGGAAGCGACATGTCGTC-3′. gRNA sequences are as follows:

gRNA1: 5′-AGATGTGCCGCTTCCAGCGG GGG-3′
gRNA2: 5′-GCTGCAGAGACAGGCGTTCG AGG-3′

### 2.8. Lentivirus Packaging and Infection

For packaging lentivirus, 4 × 10^6^ Lenti-X 293T cells (Takara, 632180, Beijing, China) were seeded in 10 cm dishes and cultured overnight to reach 70–80% confluence. Cells were transfected with 1.5 µg psPAX2 packaging plasmid (Addgene, 12260, Watertown, NY, USA), 1 µg pMD2.G envelope plasmid (Addgene, 12259), and 2 µg pLKO.1-ATG16 gRNA plasmid using Lipofectamine 2000 transfection reagent according to the manufacturer’s protocol. Briefly, plasmid DNA was diluted in 500 µL Opti-MEM (Gibco, 31985070, Thermo Fisher Scientific, Grand Island, NE, USA), and 9 µL Lipofectamine 2000 was diluted separately in 500 µL Opti-MEM. After 5 min incubation at room temperature, the diluted DNA and Lipofectamine 2000 were combined, incubated for 20 min at room temperature, and added dropwise to the cells. Medium was replaced with fresh DMEM containing 10% FBS after 6 h. The supernatant containing lentiviral particles was collected at 48 hpi, filtered through a 0.45 µm PVDF filter, and stored at −80 °C. For transfection, Vero cells were seeded in 6-well plates at 1 × 10^5^ cells per well and cultured overnight. Cells were incubated with lentiviral supernatant at a MOI of 5 in the presence of 8 µg/mL polybrene (Solarbio, H8761, Beijing, China) for 24 h and treated with 5 µg/mL puromycin (Invitrogen, A1113803) for 3 days. The positive monoclonal Vero cells were verified by DNA sequencing at Sangon Biotech (Shanghai, China). Puromycin-resistant cell pools were expanded, and knockout efficiency was validated by Western blotting as described in Section 2.5.

### 2.9. Statistical Analysis

Data are expressed as means ± standard deviation (SD). The significance of the variability between the different treatment groups was calculated by two-way analysis of variance (ANOVA) using GraphPad Prism software (version 10.0). Differences were considered statistically significant at *p* < 0.05.

## 3. Results

### 3.1. Ginsenoside Rb3 Inhibits PPRV Replication

**Figure 1:**
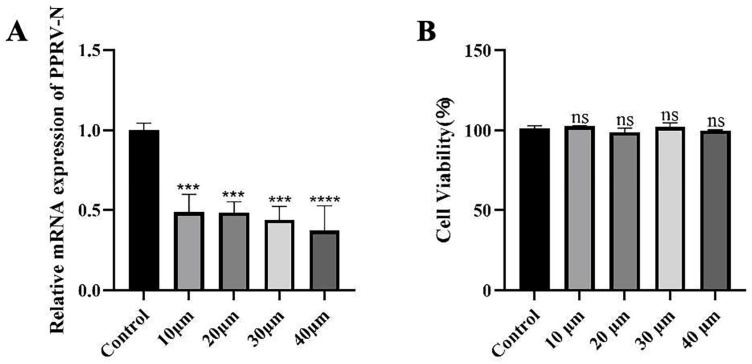
**Ginsenoside Rb3 inhibits PPRV replication in a dose-dependent manner.** (**A**) PPRV mRNA levels in various concentration Ginsenoside Rb3-treated, PPRV-infected Vero cells (MOI = 1, 48 hpi) were measured by qPCR. The data show the means ± SD; *n* = 3; *** *p* < 0.001; **** *p* < 0.0001. (**B**) Cytotoxicity of different concentration of Ginsenoside Rb3. Viability was normalized to non-treated control. The data show the means ± SD; *n* = 3; ns, no significance.

**Figure 2:**
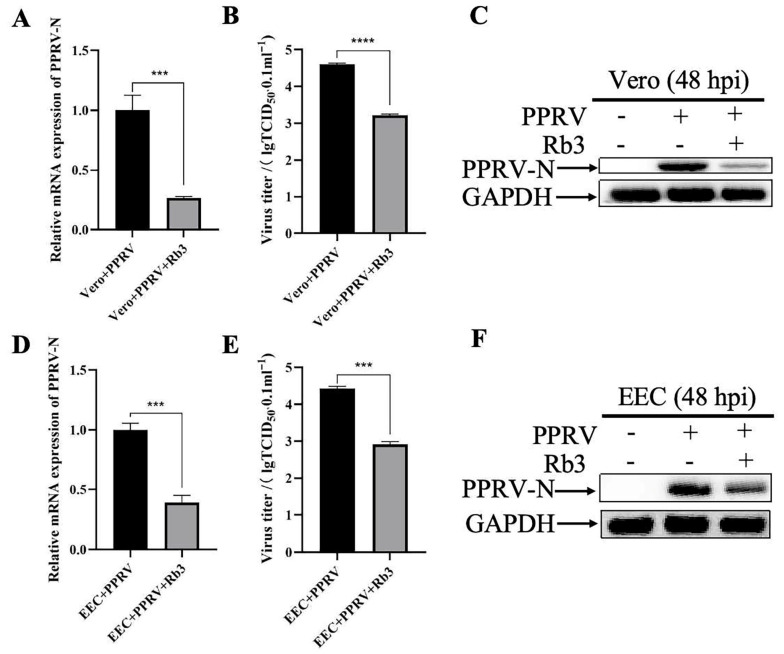
**Ginsenoside Rb3 (40 µM) significantly inhibits PPRV replication both in Vero and EEC cells.** (**A**) PPRV mRNA levels in 40 µM Ginsenoside Rb3-treated, PPRV-infected Vero cells (MOI = 1, 48 hpi) were measured by qPCR. The data show the means ± SD; *n* = 3; *** *p* < 0.001. (**B**) Control and (40 µM) Ginsenoside Rb3 pre-treated Vero cells were infected with PPRV (MOI = 1), and virus titers were measured by TCID_50_ (48 hpi). The data show the mean ± SD; *n* = 3; **** *p* < 0.0001. (**C**) Western blotting analysis of PPRV N protein in PPRV-infected wild-type and Ginsenoside Rb3-treated cells (MOI = 1, 48 hpi). GAPDH was used as a loading control. (**D**) PPRV mRNA levels in 40 µM Ginsenoside Rb3-treated, PPRV-infected EEC cells (MOI = 1, 48 hpi). The data show the means ± SD; *n* = 3; *** *p* < 0.001. (**E**) Control and (40 µM) Ginsenoside Rb3 pre-treated EEC cells were infected with PPRV (MOI = 1), and virus titers were measured by TCID_50_ (48 hpi). The data show the mean ± SD; *n* = 3; *** *p* < 0.001. (**F**) PPRV N protein level in infected wild-type and Ginsenoside Rb3-treated EEC cells (MOI = 1, 48 hpi). GAPDH was used as a loading control.

To investigate the impact of Ginsenoside Rb3 on PPRV infection, Vero cells were exposed to varying doses of Ginsenoside Rb3 for 24 h prior to viral challenge and for 48 h post-infection. At 48 hpi, quantitative real-time PCR analysis of cell lysates revealed a dose-dependent decrease in PPRV mRNA levels. While a significant decline was observed at 10 µM Ginsenoside Rb3, treatment with 40 µM resulted in an approximately 70% reduction in viral mRNA compared to the vehicle (DMEM) control (Figure 1A). This suggests that effective antiviral activity requires a relatively high concentration of Ginsenoside Rb3. Importantly, Ginsenoside Rb3 alone did not affect cell viability (Figure 1B), indicating that its inhibitory effect is not attributable to cytotoxicity. Consistently, pretreatment with 40 µM Ginsenoside Rb3 significantly lowered the PPRV mRNA level (Figure 2A,D) and infectious virus yield (Figure 2B,E) both in Vero and EEC cells and markedly reduced PPRV nucleoprotein (N) expression, as shown by Western blotting (Figure 2C,F). Together, these results demonstrate that Ginsenoside Rb3 strongly inhibits PPRV replication in vitro.

### 3.2. Ginsenoside Rb3 Inhibits PPRV Attachment, Penetration and Replication

**Figure 3:**
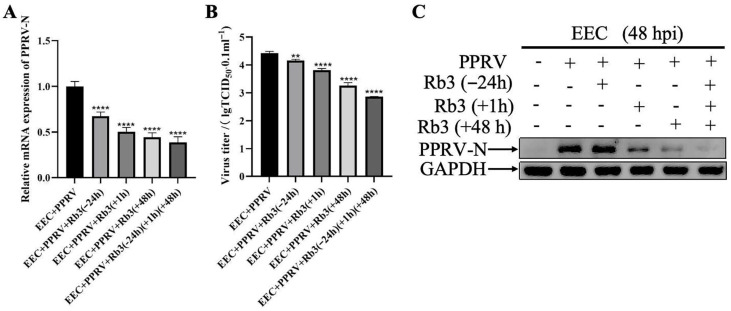
**Ginsenoside Rb3 inhibits the adsorption, invasion and replication of PPRV.** (**A**) PPRV mRNA levels in 40 µM Ginsenoside Rb3-treated, PPRV-infected EEC cells (MOI = 1, 48 hpi). The data show the means ± SD; *n* = 3; **** *p* < 0.0001. (**B**) Control and (40 µM) Ginsenoside Rb3 pre-treated EEC cells were infected with PPRV (MOI = 1), and virus titers were measured by TCID_50_ (48 hpi). The data show the mean ± SD; *n* = 3; ** *p* < 0.01; **** *p* < 0.0001. (**C**) Western blotting analysis of PPRV N protein in PPRV-infected wild-type and Ginsenoside Rb3-treated cells (MOI = 1, 48 hpi). GAPDH was used as a loading control.

We next determined on which stage in PPRV life cycle Ginsenoside Rb3 acts. To this end, EEC cells were either pretreated with 40 µM Ginsenoside Rb3 for 24 h before infection, 1 h during invasion, 48 h post infection, or both before and after infection, respectively. At 48 hpi, both PPRV RNA levels (Figure 3A) and infectious virus titers (Figure 3B) were significantly reduced in Ginsenoside Rb3-treated cells compared to the control group, as measured by RT-qPCR and TCID_50_ assay. Consistent with this, PPRV N protein levels of Ginsenoside Rb3-treated cells were markedly reduced, as shown by Western blotting (Figure 3C). The results indicated that Ginsenoside Rb3 treatment suppressed PPRV replication regardless of timing, but the inhibitory effect was more pronounced when applied post-infection, indicating that Ginsenoside Rb3 primarily inhibits PPRV replication. Collectively, these results indicate that Ginsenoside Rb3 affects multiple stages of the PPRV life cycle, including early entry events (adsorption and invasion) and subsequent replication.

### 3.3. Ginsenoside Rb3 Promotes PPRV-Induced Immune Responses

**Figure 4:**
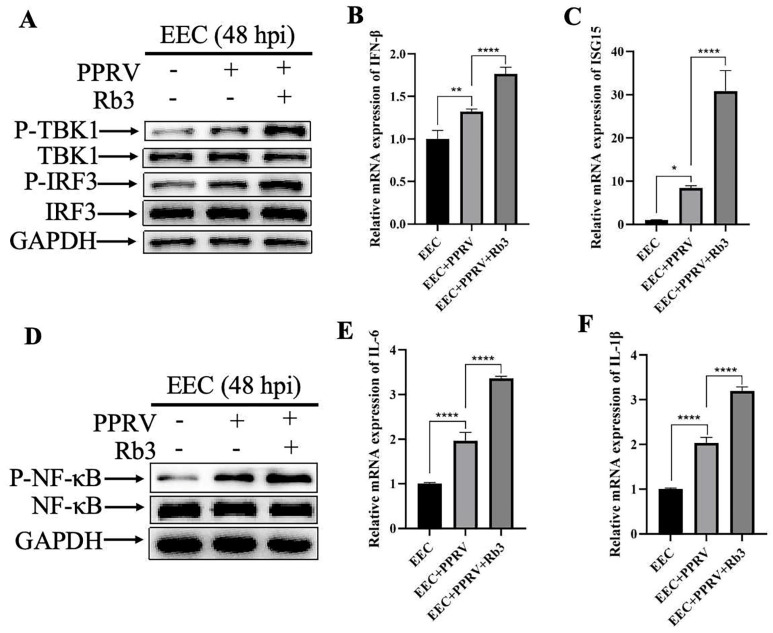
**Ginsenoside Rb3 promotes PPRV-induced immune responses.** (**A**) Western blotting analysis of P-TBK1, TBK1, P-IRF3 and IRF3 protein in PPRV-infected wild-type and Ginsenoside Rb3-treated EEC cells (MOI = 1, 48 hpi). GAPDH was used as a loading control. (**B**) IFN-β mRNA levels in 40 µM Ginsenoside Rb3-treated or not, PPRV-infected EEC cells (MOI = 1, 48 hpi). The data show the means ± SD; *n* = 3; ** *p* < 0.01; **** *p* < 0.0001. (**C**) ISG15 mRNA levels in 40 µM Ginsenoside Rb3-treated or not, PPRV-infected EEC cells (MOI = 1, 48 hpi). The data show the means ± SD; *n* = 3; * *p* < 0.1; **** *p* < 0.0001. (**D**) Western blotting analysis of P-NF-κB and NF-κB protein in PPRV-infected wild-type and Ginsenoside Rb3-treated EEC cells (MOI = 1, 48 hpi). GAPDH was used as a loading control. (**E**) IL-6 mRNA levels in 40 µM Ginsenoside Rb3-treated or not, PPRV-infected EEC cells (MOI = 1, 48 hpi). The data show the means ± SD; *n* = 3; **** *p* < 0.0001. (**F**) IL-1β mRNA levels in 40 µM Ginsenoside Rb3-treated or not, PPRV-infected EEC cells (MOI = 1, 48 hpi). The data show the means ± SD; *n* = 3; **** *p* < 0.0001.

To investigate the mechanism by which Ginsenoside Rb3 exerts its anti-PPRV activity, we assessed its impact on innate immune signaling in PPRV-infected EEC cells. Our results showed that Ginsenoside Rb3 potentiated the activation of the TBK1-IRF3 signaling axis triggered by PPRV infection in EEC cells (Figure 4A), leading to a significant upregulation of downstream antiviral effector molecules IFN-β (Figure 4B) and ISG15 (Figure 4C). Similarly, Ginsenoside Rb3 simultaneously enhanced the PPRV-dependent activation of the NF-κB pathway in EEC cells (Figure 4D) and markedly increased the expression of pro-inflammatory cytokines IL-6 (Figure 4E) and IL-1β (Figure 4F). Taken together, these results indicate that Ginsenoside Rb3 exerts its anti-PPRV activity by boosting innate immune responses.

### 3.4. Ginsenoside Rb3 Inhibits PPRV-Mediated Autophagy

**Figure 5:**
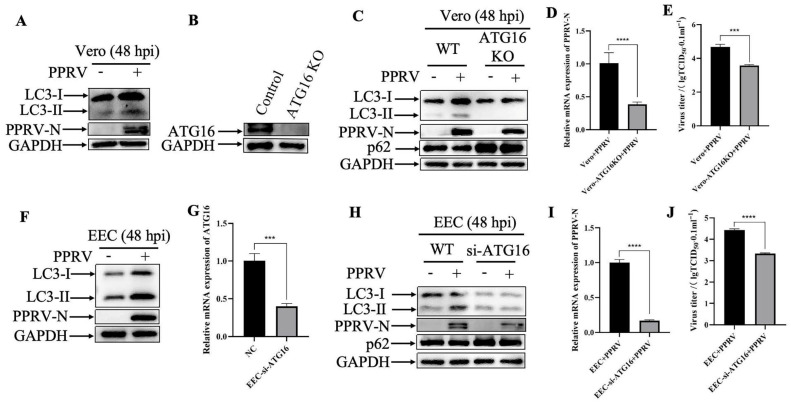
**PPRV exploits autophagy to promote its replication.** (**A**) Western blotting analysis of PPRV N protein and LC3 in PPRV-infected wild-type Vero cells (MOI = 1, 48 hpi). (**B**) ATG16 silencing efficiency were verified by Western blotting. GAPDH was used as a loading control. (**C**) Western blotting analysis of PPRV N protein, p62 and LC3 in wild-type and ATG16 KO Vero cells infected with or without PPRV infection (MOI = 1, 48 hpi). (**D**) PPRV mRNA levels in PPRV-infected wild-type and ATG16 KO Vero cells (MOI = 1, 48 hpi) were measured by qPCR. The data show the mean ± SD; *n* = 3; **** *p* < 0.0001. (**E**) Wild-type and ATG16 KO Vero cells were infected with PPRV (MOI = 1), and virus titers were measured by TCID50 (48 hpi). The data show the mean ± SD; *n* = 3; *** *p* < 0.001. (**F**) Western blotting analysis of PPRV N protein and LC3 in PPRV-infected wild-type EEC cells (MOI = 1, 48 hpi). (**G**) ATG16 mRNA levels in EEC cells transfected with ATG16-siRNA or scrambled siRNA were measured by qPCR. The data show the mean ± SD; *n* = 3; *** *p* < 0.001. (**H**) Western blotting analysis of PPRV N protein, p62 and LC3 in wild-type and ATG16 KD EEC cells infected with or without PPRV infection (MOI = 1, 48 hpi). (**I**) PPRV mRNA levels in PPRV-infected wild-type and ATG16 KD EEC cells (MOI = 1, 48 hpi) were measured by qPCR. The data show the mean ± SD; *n* = 3; **** *p* < 0.0001. (**J**) Wild-type and ATG16 KD EEC cells were infected with PPRV (MOI = 1), and virus titers were measured by TCID50 (48 hpi). The data show the mean ± SD; *n* = 3; **** *p* < 0.0001.

Autophagy is an evolutionarily conserved intracellular degradation process essential for the maintenance of cellular homeostasis through catabolic lysis of otherwise detrimental cytosolic components. In the attempt to determine the role of autophagy in the antiviral mechanism of Ginsenoside Rb3, cells were infected with PPRV, and Western blotting was performed to determine the conversion of LC3-I to LC3-II, which is currently regarded as an accurate indicator of autophagic activity. As shown in Figure 5A,F, compared with the PPRV uninfected control cells, the band intensity of LC3-II in PPRV-infected Vero and EEC cells were dramatically increased at 48 hpi, indicating that PPRV could activate autophagy in Vero and EEC cells. To further confirm the role of auphagy in PPRV replication, ATG16-deficient cells were constructed (Figure 5B,G). The results showed that the deficiency of ATG16 significantly inhibited the autophagy induced by PPRV infection (Figure 5C,H). In particular, knockout (KO) of ATG16 completely abolished the autophagy process in Vero cells. Meanwhile, loss of ATG16 dramatically decreased the expression of PPRV structural protein N (Figure 5C,H), viral mRNA levels (Figure 5D,I) and viral titers (Figure 5E,J), when compared to the PPRV-infected wild-type cells. Taken together, these findings suggest that PPRV exploits autophagy to facilitate its replication.

**Figure 6:**
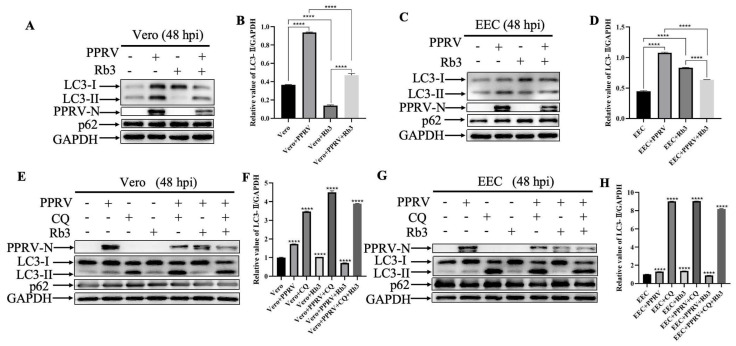
**Ginsenoside Rb3 inhibits PPRV-induced autophagy.** (**A**) Western blotting analysis of PPRV N protein, p62 and LC3 in PPRV-infected, Ginsenoside Rb3-treated (40 µM) and untreated wild-type Vero cells (MOI = 1, 48 hpi). (**B**) Gray values of Western blot bands in (**A**) were analyzed by ImageJ Version 1.8.0, and LC3-II values were normalized to GAPDH intensity. The data show the mean ± SD; *n* = 3; **** *p* < 0.0001. (**C**) Western blotting analysis of PPRV N protein, p62 and LC3 in PPRV-infected, Ginsenoside Rb3-treated (40 µM) and untreated wild-type EEC cells (MOI = 1, 48 hpi). (**D**) Gray values of Western blot bands in (**C**) were analyzed by ImageJ Version 1.8.0, and LC3-II values were normalized to GAPDH intensity. The data show the mean ± SD; *n* = 3; **** *p* < 0.0001. (**E**) Western blotting analysis of PPRV N protein, p62 and LC3 in PPRV-infected, Ginsenoside Rb3 (40 µM) and/or Chloroquine (50 µM)-treated and untreated wild-type Vero cells (MOI = 1, 48 hpi). (**F**) Gray values of Western blot bands in (**C**) were analyzed by ImageJ Version 1.8.0, and LC3-II values were normalized to GAPDH intensity. The data show the mean ± SD; *n* = 3; **** *p* < 0.0001. (**G**) Western blotting analysis of PPRV N protein, p62 and LC3 in PPRV-infected, Ginsenoside Rb3 (40 µM) and/or Chloroquine (50 µM)-treated and untreated wild-type EEC cells (MOI = 1, 48 hpi). (**H**) Gray values of Western blot bands in (**C**) were analyzed by ImageJ Version 1.8.0, and LC3-II values were normalized to GAPDH intensity. The data show the mean ± SD; *n* = 3; **** *p* < 0.0001.

To further validate the impact of Ginsenoside Rb3 on PPRV-induced autophagy, cells were infected with PPRV at MOI of 1 for 48 h with the treatment of 40 µM Ginsenoside Rb3. Our results showed that treatment with Ginsenoside Rb3 alone had no effect on cellular basal autophagy. However, Ginsenoside Rb3 markedly inhibited the autophagy process triggered by PPRV infection both in Vero (Figure 6A,B) and EEC cells (Figure 6C,D). To gain insight into autophagic flux, inhibitors Chloroquine (50 µM) was applied for 6 h prior to infection. Our data shown that inhibition of autophagy with Chloroquine facilitate the Ginsenoside Rb3-dependent blockage of autophagy during PPRV infection both in Vero (Figure 6E,F) and EEC cells (Figure 6G,H), evidenced by increased p62 and LC3-II levels, suggesting impaired autophagic flux. As shown, Chloroquine treatment alone inhibited autophagy (decreased LC3-II degradation) in uninfected cells, consistent with its known function. In PPRV-infected cells, Chloroquine treatment decreased virus-induced autophagy and significantly decreased PPRV replication. Importantly, when Ginsenoside Rb3 was combined with Chloroquine in PPRV-infected cells, Ginsenoside Rb3 significantly attenuated Chloroquine-induced autophagy diminish and promoted the anti-viral effect of Chloroquine. In summary, these findings imply that the anti-PPRV activity of Ginsenoside Rb3 is attributable to its ability to inhibit PPRV-induced autophagy.

### 3.5. Inhibition of Autophagy with Ginsenoside Rb3 Promotes PPRV-Mediated Immune Responses

**Figure 7:**
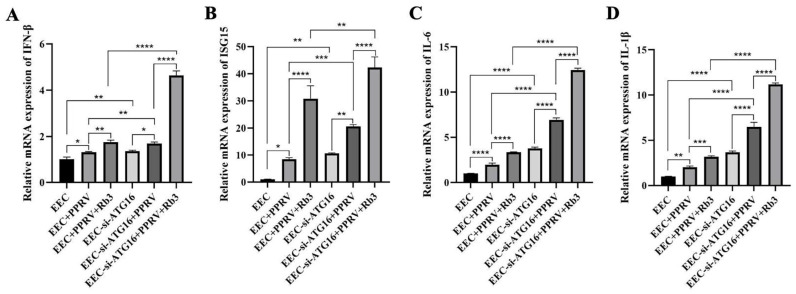
**Ginsenoside Rb3 suppresses PPRV-induced autophagy to promote immune responses.** (**A**) IFN-β mRNA levels in 40 µM Ginsenoside Rb3-treated or not, PPRV-infected wild-type and ATG16 KD EEC cells (MOI = 1, 48 hpi). The data show the means ± SD; *n* = 3; * *p* < 0.1; ** *p* < 0.01; **** *p* < 0.0001. (**B**) ISG15 mRNA levels in 40 µM Ginsenoside Rb3-treated or not, PPRV-infected wild-type and ATG16 KD EEC cells (MOI = 1, 48 hpi). The data show the means ± SD; *n* = 3; * *p* < 0.1; ** *p* < 0.01; *** *p* < 0.001; **** *p* < 0.0001. (**C**) IL-6 mRNA levels in 40 µM Ginsenoside Rb3-treated or not, PPRV-infected wild-type and ATG16 KD EEC cells (MOI = 1, 48 hpi). The data show the means ± SD; *n* = 3; **** *p* < 0.0001. (**D**) IL-1β mRNA levels in 40 µM Ginsenoside Rb3-treated or not, PPRV-infected wild-type and ATG16 KD EEC cells (MOI = 1, 48 hpi). The data show the means ± SD; *n* = 3; ** *p* < 0.01; *** *p* < 0.001; **** *p* < 0.0001.

As PPRV make use of autophagy to promote its replication, abolition of autophagy by ATG16 knockout would lead to a lower viral yield than that of ATG16 knockdown cells, which only partly blocked autophagy flux. However, according to our observations, PPRV N protein levels (Figure 5C), PPRV mRNA levels (Figure 5D) and virus titers (Figure 5E) in ATG16 KO Vero cells were all higher than those in ATG16 KD EEC cells (Figure 5H–J). Considering Vero cells have a genetic defect in interferon production, we thus speculated that autophagy may have an effect on PPRV-triggered interferon production. To test this hypothesis, we measured the interferon levels both in wild-type and ATG16 KD EEC cells. As shown, compared with PPRV-infected wild-type EEC cells, the mRNA levels of IFN-β (Figure 7A) and ISG15 (Figure 7B) were significantly increased in ATG16 defective cells. What is more, the promotion effect of Ginsenoside Rb3 on IFN-β and ISG15 production in PPRV-infected ATG16 KD cells were stronger than that in PPRV-infected wild-type EEC cells (Figure 7A,B), indicating that Ginsenoside Rb3 exerts its anti-PPRV activity by enhancing PPRV-mediated interferon responses through autophagy inhibition. Meanwhile, defectiveness of ATG16 increased pro-inflammatory cytokines IL-6 (Figure 7C) and IL-1β (Figure 7D) production as well. Furthermore, Ginsenoside Rb3 had a similar effect on these pro-inflammatory cytokines production (Figure 7C,D) when compared with its promoting effect on interferon responses, the promotion effect of Ginsenoside Rb3 on IL-6 and IL-1β production in PPRV-infected ATG16 KD cells were stronger than that in PPRV-infected wild-type EEC cells. Taken together, these findings suggest that the antiviral activity of Ginsenoside Rb3 is due to its inhibitory effect on PPRV-induced autophagy resulted in potent promotion of innate immune responses.

## 4. Discussion

PPRV poses a severe threat to global small ruminant populations due to its high contagion and mortality rates [1], having already incurred substantial economic losses in endemic regions [55]. Currently, vaccination represents the primary strategy for controlling PPRV transmission. Live attenuated vaccines are widely deployed and serve as cornerstone tools in international eradication efforts [56,57]. Nevertheless, their effectiveness is constrained by thermolability—a characteristic shared among paramyxoviruses—necessitating stringent cold-chain logistics, especially in warmer climates, which substantially escalates distribution costs [58]. These limitations underscore the pressing demand for the development of antiviral therapeutics capable of complementing or supplementing vaccination. To date, however, no antiviral agents specifically targeting PPRV have been reported, highlighting a critical gap in the current management.

Ginseng is a medicinal herb sourced from the roots of several species, most notably Panax ginseng (Korean/Asian ginseng), Panax quinquefolius (American ginseng), and Eleutherococcus senticosus (Siberian ginseng) [59]. It has been widely employed in traditional medicine systems for its purported benefits in modulating immune responses and enhancing physical performance [37]. Ginseng is predominantly administered orally. Ginsenosides are considered the major components of ginseng, following ingestion, intestinal microbial flora metabolize major ginsenosides into bioactive derivatives [35,60]. Fermented ginseng preparations have demonstrated a range of physiological effects, such as antioxidant and antibacterial properties [61], mitigation of dextran sulfate sodium-induced colitis [30], and antidiabetic activity [31]. Several Ginsenosides have demonstrated antiviral properties across different viral models. 20(R)-ginsenoside Rh2 suppressed gamma herpesvirus replication in murine and human systems [62], while 20(S)-protopanaxtriol exhibited potent activity against coxsackievirus B3 (CVB3) in vitro [63]. Ginsenosides Re, Rf, and Rg2 conferred protection against rhinovirus 3 and coxsackievirus infections. Additionally, Rg2 has shown significant anti-Enterovirus 71 (EV71) activity [33]. Ginsenoside Rg3 inhibited HCV-induced persistent infection [32]. Furthermore, Rb2 reduced viral titers and provided protection against rotavirus and Sendai virus infections in mice [64,65]. Ginseng extracts ihibited a broad-spectrum of influenza viruses [35]. The findings by Kang et al. [36] established Ginsenoside Rb1 as an immunostimulatory agent with antiviral efficacy against EV71 in both cellular and animal models. Ginsenoside Rb3 demonstrates therapeutic potential against Pestivirus infections, including BVDV and CSFV [34]. In line with this, our study demonstrates that Ginsenoside Rb3 also exerts significant inhibitory effects on PPRV replication in vitro. While our experiments demonstrate that Ginsenoside Rb3 inhibits early stages of infection (adsorption and invasion), the precise mechanism—whether through direct interaction with viral particles, modulation of host receptors, or interference with membrane fusion—remains to be determined.

The control of pathogenic microbes involves a coordinated immune response, wherein innate immunity acts as the primary barrier, followed by pathogen-specific clearance mediated by the adaptive system [66,67]. A cornerstone of innate defense is the IFN response, which rapidly induces the expression of key immune mediators such as cytokines and chemokines, forming the essential early layers of host protection. Ginseng is widely used as herbal tonic due to its regulation of immune function [68]. Studies have shown that Ginsenoside Rb1 can enhance both cellular and humoral immune responses in vivo and potentiate IFN signaling. Knockdown of IFN-β abolished the antiviral effect of Ginsenoside Rb1 [34,36]. Furthermore, Rb1 treatment has been reported to upregulate IFN-α, IFN-β, and the IFN-induced protein MxA in specific viral infection models [33,69,70]. While Ginsenoside Rh1 is recognized for its anti-inflammatory effects on ameliorating asthma in mice by restoring Th1/Th2 cytokine balance [37,71]. Studies have shown that ginseng extracts attenuate the release of pro-inflammatory cytokines IL-6 and IL-8 and stimulate interferon (IFN)-β expression in mice challenged with influenza virus [72,73]. Tao Yu et al. showed that Ginsenoside molecules markedly suppress the expression of cytokines, such as TNF-α and IL-1 [74]. Our results demonstrate that Ginsenoside Rb3 enhances the PPRV-induced pro-inflammatory cytokine response alongside its potent stimulation of the TBK1-IRF3 axis and type I interferon signaling, which is in contrast to Ginsenosides known anti-inflammatory properties. This suggests that the primary role of Ginsenosides may not be uniformly anti-inflammatory but rather that of immune balancers and contextual immunomodulators. The function of Ginsenosides appears to be highly dependent on the host’s existing immune status and the nature of the pathogenic challenge. In the context of a viral infection like PPRV, where a robust early innate response is crucial, Ginsenosides Rb3 may prioritize amplifying essential antiviral defenses.

Autophagy is an evolutionarily conserved lysosomal degradation pathway essential for maintaining cellular homeostasis and eliminating intracellular pathogens [75]. While autophagy can serve as an antiviral defense mechanism by directly degrading viral components, delivering viral nucleic acids to pattern recognition receptors, and supporting antigen presentation [76], on the other hand, numerous viruses [77], including MV [78], HCV [79], foot-and-mouth disease virus (FMDV) [80], influenza A virus (IAV) [81] and dengue virus (DENV) [82], have evolved mechanisms to manipulate autophagy to evade immune clearance and promote their own replication. Similar to MV, PPRV, a morbillivirus closely related to MV, has been shown to hijack the autophagic machinery to facilitate its replication [15,54]. Our study confirmed that PPRV infection activates autophagy and inhibition of autophagy significantly decreased the viral replication, emphasizing the importance of autophagy during PPRV infection.

Autophagy serves not only as a fundamental cellular defense mechanism that eliminates microbial pathogens through autophagosomal degradation but also plays a pivotal role in initiating both innate and adaptive immune responses [50,83,84]. It has been reported that ATG13 exerts antiviral activity against PPRV by enhancing IFN production and depletion of ATG13 markedly diminished the capacity of RIG-I to activate IFN responses [85]. Conversely, we found that genetic inhibition of autophagy by silencing ATG16 upregulated both antiviral mediators (IFN-β and ISG15) and pro-inflammatory cytokines (IL-1β and IL-6). We acknowledge that the current study lacks rescue experiments (e.g., restoring autophagy with rapamycin or ATG16 overexpression) that would establish a causal relationship between autophagy inhibition and enhanced interferon responses. It has been demonstrated that epithelial ATG16-mediated autophagy in IECs serves as an inflammatory hub driving metabolic enteritis [86]. On the other hand, ATG16 is not exclusively dedicated to autophagy; it has been implicated in other cellular processes, including regulation of immune signaling and inflammasome activity [87]. Therefore, the enhanced IFN responses observed in ATG16-deficient EECs cells could reflect loss of these non-autophagy functions rather than, or in addition to, inhibition of autophagic flux. Future studies using multiple autophagy-targeting approaches are needed to dissect the autophagy-specific contribution to the observed immunomodulatory effects. All experiments in this study were performed using the attenuated vaccine strain PPRV Nigeria 75/1, as virulent PPRV strains were not available to us. While this represents a limitation, studies on the related measles virus have shown that both wild-type and vaccine strains can induce autophagy, albeit through distinct molecular pathways [78]. Furthermore, the well-documented ability of PPRV to actively suppress IFN-β induction via its C protein [88,89] provides a relevant context for our findings that Rb3 enhances IFN responses. The enhanced immune responses observed could be, at least in part, a consequence of the inhibition of autophagy resulting from Ginsenoside Rb3’s antiviral activity. Nevertheless, future studies using virulent PPRV strains would be valuable to confirm whether the observed effects of Rb3 on autophagy and innate immunity translate to pathogenic infection models.

Ginsenosides, including Rb3, are known to have limited oral bioavailability due to poor membrane permeability, extensive metabolism by gut microbiota, and rapid elimination. While 40 µM Rb3 was non-cytotoxic in our cell models, we cannot exclude the possibility of off-target effects on cellular pathways beyond autophagy and innate immune signaling. Typical peak plasma concentrations of ginsenosides after oral administration in rodents or humans are in the low micromolar or nanomolar range. The effective concentration of Ginsenoside Rb3 identified in this study (40 µM) is relatively high, and achieving such concentrations systemically through oral administration would be challenging. Some ginsenosides have been shown to accumulate in specific tissues [90,91] at concentrations higher than those in plasma. It is possible that local concentrations at sites of PPRV replication (e.g., respiratory and gastrointestinal epithelia) could approach the effective range.

It is important to acknowledge that the present study was conducted exclusively using in vitro cell culture systems. While these models are valuable for dissecting molecular mechanisms and providing initial evidence of antiviral activity, they do not fully recapitulate the complex physiological environment of a living host. Factors such as drug absorption, distribution, metabolism, excretion, and potential toxicity—all of which critically influence in vivo efficacy—cannot be assessed in cell-based assays. Moreover, for naturally occurring compounds like Ginsenoside Rb3, additional complexities arise from metabolism by gut microbiota, potential interactions with the host immune system in its full complexity, and the need to achieve therapeutically relevant concentrations at sites of viral replication. Future studies employing suitable animal models of PPRV infection are essential to determine whether the antiviral effects observed in vitro translate into clinically meaningful outcomes.

## 5. Conclusions

In conclusion, we demonstrated for the first time that Ginsenoside Rb3 effectively inhibits PPRV replication in vitro. Evaluation of the mechanism of action of Ginsenoside Rb3 against PPRV revealed that the antiviral activity is due to the stimulation of immune responses by inhibiting PPRV-mediated autophagy, highlighting that modulation of autophagy represents an attractive approach to counteract viruses. Since Ginsenoside Rb3 is a clinically widely used herbal medicine, our data might provide a new therapeutic option for the cure and possibly also the prevention of other related Morbillivirus infection.

## Data Availability

The original contributions presented in this study are included in the article. Further inquiries can be directed to the corresponding author.

## Abbreviations

The following abbreviations are used in this manuscript:

PPR	Peste des petits ruminants
PPRV	Peste des petits ruminants virus
RPV	Rinderpest virus
CDV	Canine distemper virus
PDV	Phocine distemper virus
DMV	Dolphin morbillivirus
PMV	Porpoise morbillivirus
MV	Measles virus
HCV	Hepatitis C virus
BVDV	Bovine viral diarrhea virus
CSFV	Classical swine fever virus
RIG-I	Retinoic acid-inducible gene I
MDA5	Melanoma differentiation-associated gene 5
MAVS	Mitochondrial antiviral signaling protein
TBK1	TANK-binding kinase 1
IRF3	Interferon regulatory factor 3
IFN	Interferon
ISG	Interferon-stimulated gene
IKK	IkB kinase
NF-kB	Transcription factors of the nuclear factor kB
IL-6	Interleukin-6
TNF-α	Tumor necrosis factor-α
ATG	autophagy-related gene
LC3	Microtubule-associated protein 1 light chain 3
EV71	Enterovirus 71
FMDV	Foot-and-mouth disease virus
IAV	Influenza A virus
